# Eight Indicators for Measuring Equitable Student Success in STEM

**DOI:** 10.1080/00091383.2024.2348425

**Published:** 2024-06-18

**Authors:** Kacy Redd, Mica Estrada, Harriet B. Nembhard, Courtney Ngai

**Affiliations:** Association of Public and Land-Grant Universities (APLU); University of California; Harvey Mudd College; Undergraduate Research and Artistry at Colorado State University

In an era where STEM expertise is desperately needed to help solve critical world challenges, ensuring the success of students in STEM, particularly those who have been historically marginalized, underserved, and/or excluded, has never been more pressing. In response to this challenge, the authors, informed by the work of the National Academies for Sciences, Engineering, and Medicine (NASEM) Roundtable on Systemic Change in Undergraduate STEM Education (NASEM, n.d.), offer key guidance for educators from 2-year and 4-year colleges and universities who want to better assess equitable student success in STEM. While we have focused on STEM in this article, this guidance can be applied to all disciplines, academic programs, and institution-wide efforts.

In this article, we provide four reflective questions and eight measurable indicators to help guide the development and refinement of how equity is being measured. The questions serve as a compass for institutions seeking to better support their STEM students. We provide indicators and evidence for each question drawn from NASEM’s *Indicators for Monitoring Undergraduate STEM Education* (NASEM, 2018). Our goal is to offer a concise, targeted resource that fuels data-informed conversations within your academic program, department, college, or university regarding STEM student support.

For this article, we define equitable student success as fostering diversity, nurturing an equitable and inclusive learning environment, cultivating a sense of belonging, and actively eliminating bias in student outcomes. This definition and the associated indicators build on the data required by the U.S. Department of Education (e.g., student admissions, enrollment, retention rate, and graduation rate; Integrated Postsecondary Education Data System, n.d.) and emphasize the importance of additional data and evidence for a more holistic assessment of student success. We invite institutional leaders at all levels to embrace our broader definition of equitable student success and to cultivate a culture of data collection and use to ensure equitable outcomes for students pursuing STEM fields.

A key principle underlying this guidance is that institutions should analyze student experiences and student outcomes by examining the data and evidence that are available at their institution. Where feasible, this evidence can be disaggregated by demographic categories standardized across institutional units (e.g., admissions, departments, and financial aid offices). Further, we urge institutions to explore these data at the intersections of race, ethnicity, gender, and other demographic characteristics, where possible. If institution-wide data are not available for an indicator, an institution can consider a smaller-scale data collection activity and/or conduct a focus group with faculty and students. While there is no one-size-fits-all approach to data disaggregation, institutions may consider demographic categories including, but not limited to, race, ethnicity, gender, gender expression, sexual orientation, religion, language, geographic region, ability status, parenting status, socioeconomic status, and veteran status, as well as whether students are full time or part time, first generation, or have transferred for their data collection. Intersections of these categories are important to examine where possible while protecting student privacy. Institutions can weigh which of these categories are available for analysis, most salient for their student populations, and may be most relevant for examining equity gaps (i.e., in enrollment, STEM graduation rates, access to research experiences, or time-to-degree disparities).

Additionally, a cross-campus approach with an integrated series of supports is important. Leaders, including presidents, provosts, vice provosts of undergraduate education, executive vice presidents and vice presidents of instruction, vice presidents of student affairs, and deans, provide powerful and meaningful leadership when they champion the collection and use of student data for program improvement and appropriately resource these efforts. Through collaboration with other stake-holders, they can establish institutional targets for program outcomes to increase accountability, celebrate success, and identify areas for future growth.

As research has amply demonstrated, a top-down approach alone is not sufficient (Holcombe et al., 2021). An effective collaborative approach requires the engagement of institutional data stewards or institutional research officers, directors of student success who have data on student engagement beyond the classroom, department leaders entrusted with overseeing undergraduate courses, Centers for Teaching and Learning, and STEM Education Center staff (Carlisle & Weaver, 2018, 2020; Horii et al., 2015; Wright et al., 2018) who can help facilitate reflective dialog (Kruse & Louis, 1993) and implementation of evidence-based and inclusive pedagogical practices. Department chairs and faculty, including Visiting faculty, Instructors, Teaching Assistants, teaching professors, adjunct faculty, and Lecturers or VITAL faculty (Lee et al., 2023; Levy, 2019), are central to this effort. STEM faculty interact most closely with their STEM students as classroom instructors, research and career mentors, and often as academic advisors. Finally, campuses would benefit from including students in these reflective dialogs to ensure that the desired outcomes of the STEM students are being met (Ellis-Nelson et al., 2023). These dialogs can also be an educational opportunity for students to build their data expertise.

## Reflective Questions, Indicators, and Evidence

These reflective questions and eight indicators provide a structured approach for institutions to identify areas for improvement and to support student success effectively. The indicators have been adapted from NASEM’s *Indicators for Monitoring Undergraduate STEM Education* (2018); we indicate the alignment of each indicator to this report in brackets. Undoubtedly, different institutions will focus on different indicators and types of evidence that are best aligned with their institutional mission, available resources, student populations served, and local context.

### Reflective Question 1: Does your institution, college, and/or STEM department/academic program encourage a diverse and equitable enrollment, and establish accessible and supportive entry pathways for all potential students, particularly into STEM programs?

Indicator 1: Diversity of enrollees in STEM programs compared to the diversity of enrollees across academic fields. [Aligned with Indicators 2.2.1 and 2.2.2]

Evidence:

- If available, review historical data showing trends in the diversity of students enrolled in STEM versus non-STEM degree or certificate programs over a specified period.
- Examine disaggregated demographic data showing the percentages of students of differing race, ethnicity, gender, transfer status, first-generation status, socioeconomic status, and other relevant backgrounds and identities who enroll in STEM certificate and degree programs versus those enrolled in non-STEM programs. These data should allow for an intersectional analysis.

Indicator 2: Outcomes of STEM transfer students compared to all transfer students (STEM and non-STEM programs at the institution). [Aligned with Indicator 3.2.2]

Evidence:

- Students are increasingly transferring between institutions. Regardless of the transfer path, determine the ratio of students who transfer into the institution and declare a STEM-related major to the total number of students who transferred to the institution.
- Evaluate the acceptance rate of transfer credits, especially in STEM fields, for incoming transfer students. Disaggregate these data for deeper analysis.
- Compare the performance of transfer students in courses where they completed the prerequisite at a different institution with students who completed the prerequisite at their current institution.
- Compare the use of support services, such as advising, orientation programs, and learning communities, by STEM transfer students to all transfer students and to all STEM students.

### Reflective Question 2: Does your institution, college, and/or STEM department/academic program develop equitable learning environments that foster learning for all students in STEM programs?

Indicator 3: Extent of the implementation of evidence-based educational practices in STEM courses. [Aligned with Indicator 1.1.1]

Evidence:

- Gather survey data on student perceptions of the learning environment, including their engagement and satisfaction in STEM courses.
- Evaluate whether resources, rewards, and accountability systems are structured to encourage and sustain instructors’ use of evidence-based practices (TEval, n.d.). Look for indications of institutional recognition programs for teaching, funds allocated for faculty professional development in teaching, and usage of validated measures (such as observation protocols and teaching portfolios) that go beyond just student evaluations to assess teaching performance. Evidence may include the following:
  - Percentage of instructors (tenure track and nontenure track) who report annual engagement in education-related professional development.
  - Average funding available for instructional development and support per instructor.
  - Percentage of instructors who report using evidence-based instructional practices to support student learning.
  - Percentage of instructors who are observed to use evidence-based instructional practices as documented by observation protocols such as Classroom Observation Protocol for Undergraduate STEM (Smith et al., 2013), Reformed Teaching Observation Protocol (RTOP; Sawada et al., 2002), Teaching Dimensions Observation Protocol (Hora et al., 2013), and Practical Observation Rubric to Assess Active Learning (PORTAAL; Eddy et al., 2015).
- Assess the extent to which departments consider the use of evidence-based teaching in decisions for hiring, merit, retention, and promotion.

Indicator 4: Extent to which there is equitable student access to and participation in evidence-based educational programs and experiences. [Aligned with Indicator 2.1.3]

Evidence:

- Determine if the institution or department has clearly defined minimum criteria for educational experiences and methods for monitoring access to these experiences (Estrada, 2014). Key data points could include the following:
  1. Percentage of students who have engaged in an experiential learning experience such as many of the Association of American Colleges & Universities’s High Impact Practices (2023; Kuh, 2008) or other authentic experiential learning experiences as defined by the institution. Examples include course-based research experiences, National Science Foundation’s Community College Undergraduate Research Initiative (n.d.), and first-year research initiatives aimed at ensuring every STEM student has access to an authentic research experience.
  2. Percentage of students who have an assigned mentor with whom they meet regularly.
  3. Percentage of departmental and/or institutional funds allocated for this work and/or for the students to engage in these opportunities.

### Reflective Question 3: Does your institution, college, and/or STEM department/academic program foster a sense of inclusion and belonging for all students in your program?

Indicator 5: Extent to which students feel included and have a sense of belonging in their STEM academic programs. [Aligned with Indicator 2.4.1]

Evidence:

- Assess the extent to which the curriculum in STEM programs is inclusive and culturally responsive by reviewing the curriculum and syllabi to identify the inclusion of diverse perspectives, theories, or applications in STEM (Estrada et al., 2018; Student Experience Project, 2022).
- Evaluate the accessibility of learning materials and resources by reviewing course materials and learning resources for accessibility (considering disabilities, language proficiency, etc.).
- Disaggregate data on the recipients of financial support to enable intersectional analysis.
- Evaluate the extent to which classrooms are inclusive and support diversity through surveys or focus groups to measure students’ perceptions of classroom inclusivity.
- Survey students on their satisfaction, sense of belonging, and perspectives, which could include regularly conducting climate surveys on students’ belonging, support systems, and access to resources within their program and/or institution.
- Investigate faculty and staff professional development efforts focused on fostering an inclusive and supportive classroom environment.

Indicator 6: Extent to which students pursuing STEM credentials and degrees persist in STEM programs, from course to course, and year to year. [Aligned with Indicators 2.1.2 and 3.2.1]

Evidence:

- Track incoming students’ degree aspirations and the declaration of STEM degrees through surveys or other measures capturing the specific STEM fields incoming students intend to pursue. Analyze trends over time in degree aspirations among different demographic groups.
- Calculate the number of students, disaggregated by demographic characteristics, who switch their declaration away from a STEM major, and at what point in their academic career this occurs.
- Investigate barriers or challenges faced by students who switch their declaration away from a STEM major, such as course availability, academic advising, or particular courses that have high Drop/Fail/Withdraw rates.
- Monitor if students stay in their declared STEM major, their course load per semester, Grade Point Average, and progression/grades in subsequent courses.
- Track the success rates (grades, class completions) of STEM students over time, disaggregated by demographic categories and other relevant factors.
- Review student support services usage data (tutoring, academic advising, mental health services) to see if there is a correlation between service usage and retention in STEM programs.

### Reflective Question 4: Does your institution, college, and department/academic program ensure equitable outcomes for all students, particularly in STEM programs?

Indicator 7: Percentage of students across demographic categories who complete a credential or degree in STEM compared to the percentage of students who complete a credential or degree across academic fields. [Aligned with Indicator 3.3.1]

Evidence:

- Compare the graduation rates of STEM students with the institution’s overall graduation rates to identify potential gaps or disparities. Examine the data disaggregated by demographic categories and attend to relevant and available intersectional analyses.
- Monitor the number and percentage of students who complete a STEM degree or certificate program.
- For students who begin in a 2-year program and transition into a 4-year program, track their degree completion rates compared to 4-year-only students.
- Examine postgraduation metrics, such as job placements in STEM fields, graduate-school acceptances, and other indicators of success in the field.

Indicator 8: Time-to-degree for students in STEM programs compared to time-to-degree for students across academic programs, disaggregated by demographic categories and other relevant factors. [Aligned with Indicator 2.2.3]

Evidence:

- Calculate the time required for students to earn their STEM degrees, measured by time to degree at 100 percent (2 years for 2-year institutions or 4 years for 4-year institutions), 150 percent, and 200 percent. Disaggregate the data by race, ethnicity, gender, transfer status, first-generation status, socioeconomic status, and other relevant characteristics. Assess whether there is a correlation between longer time-to-degree and other factors, such as part-time enrollment, work commitments, or family responsibilities.
- Calculate the average academic terms (semesters or quarters) needed to obtain a STEM degree or credential, disaggregated by relevant factors.

## Final Remarks

We invite institutional leaders at all levels to champion a culture of smart data use in pursuit of equitable student success. The reflective questions, indicators, and evidence outlined in this document serve as a guide for your institution and/or department to assess how well you are serving STEM students. As a starting place for this transformative journey, NASEM recommended bench-marking the demographic representation of STEM undergraduate degree/certificate earners compared to the demographic representation of all undergraduate graduates during the same period within the institution. NASEM recommended this comparison group because these students have successfully navigated higher education. However, this is merely a benchmark; your institution, college, department, or academic program may want to benchmark against peer institutions or your institution may want to choose a more ambitious target that defines successfully and equitably serving all students.

## Figures and Tables

**Figure 1.**
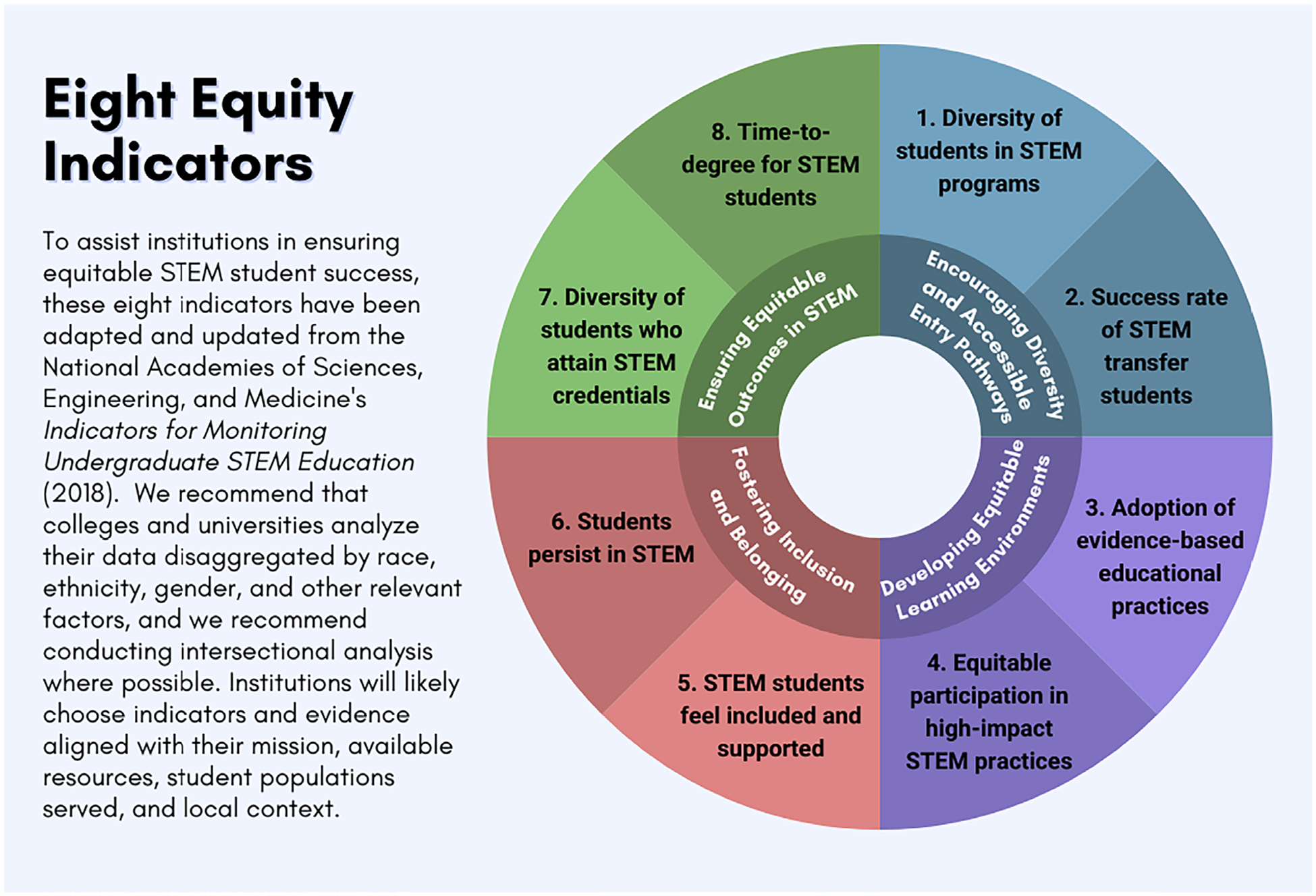
Eight Equity Indicators for Institutions to Ensure Equitable Stem Student Success, Adapted from the National Academies of Sciences, Engineering, and Medicine (2018)

## In Short

- Institutions can begin to answer if they have a diverse enrollment and accessible entry pathways by examining (1) the diversity of STEM enrollees and (2) the success rate of STEM transferees, disaggregated by relevant demographic factors.
- They can assess the (3) adoption of evidence-based educational practices and (4) equitable participation in high-impact STEM practices to know if they have equitable learning environments that foster learning for all students.
- Institutions have evidence that they are fostering inclusion and belonging for all STEM students if (5) STEM students report feeling included and supported and (6) students persist in STEM.
- Finally, institutions can know that they are ensuring equitable outcomes if (7) there are no equity gaps in the percentage of students who attain STEM credentials or (8) longer time-to-degree for diverse STEM students (see Figure 1).

## An Example of These Reflective Questions in Action

To effectively engage with the reflective questions, one valuable approach is to use the Partnership for Undergraduate Life Sciences (PULSE) tools (Brancaccio-Taras et al., 2016; PULSE Rubrics, 2023). During the 2022–2023 academic year, the University of Iowa College of Engineering embarked on a journey, prompted by then-dean Nembhard, to discuss and assess the current state of affairs within each department (Vigmostad, 2023). Specifically, they were tasked with assessing their department’s current consensus score for the 13 criteria on the PULSE Diversity, Equity, and Inclusion (DEI) Rubric. This rubric encompasses four distinct categories:

- Curriculum (aligned with Reflective Question 1).
- Assessment (aligned with Reflective Question 2).
- Faculty Practice and Support (aligned with Reflective Questions 2 and 3).
- Climate for Change (aligned with Reflective Question 4).

To facilitate this assessment, faculty discussion of the PULSE DEI Rubric occurred at departmental faculty meetings led by the department chairs or the chair of the department’s DEI committee. These discussions were useful in guiding the faculty to the critical juncture where *they sought* the empirical data required to address the reflective questions effectively, resulting in a data-driven approach to enhancing DEI within the College of Engineering.

## Additional Resources and Tools

- The National Academies of Sciences, Engineering, and Medicine have three reports that will be useful for readers: *Barriers and Opportunities for 2-year and 4-year STEM Degrees* (2016); *Indicators for Monitoring Undergraduate STEM Education* (2018); and *Advancing Antiracism, Diversity, Equity, and Inclusion in STEMM Organizations* (2023).
- The Sloan Equity and Inclusion in STEM Introductory Courses (SEISMIC) Equity Learning Communities Report (n.d.) outlines how demographic student data might be analyzed for insights into course outcomes, grade distribution patterns, and grade anomalies. They provide source code via GitHub so that institutions can run their analysis.
- The Association of Public and Land-Grant Universities’s *Data Maturity Index* has questions and criteria that can help institutional leaders evaluate how advanced their institutional data culture is for data governance, quality, analysis, dissemination, and application (Powered by Publics, 2023).
- The National Institutes of Health and the Howard Hughes Medical Institute convened the *Joint Working Group on Improving Underrepresented Minorities (URMs) Persistence in STEM*, which made five recommendations for institutions to increase URM persistence in STEM (Estrada et al., 2016). The first recommendation was to track success and failure and collect data to increase institutional accountability.
- There is a growing body of research on how to support departmental change that includes departmental change principles (Quan et al., 2019; Reinholz et al., 2017), frameworks (Corbo et al., 2016; Reinholz & Apkarian, 2018), and approaches (Andrews et al., 2021; Ngai et al., 2020; Reinholz et al., 2017, 2019).
- In addition to the department-level resources from PULSE (PULSE Rubrics, 2023) in the life sciences, the physics community has also developed a comprehensive set of resources for physics departments in their *Effective Practices for Physics Programs (EP3) Guide and Toolkits* for engaging in systemic improvement (EP3 Guide, 2023).
